# Association between sinusitis and relapse and changes in the myeloperoxidase–antineutrophil cytoplasmic antibody in microscopic polyangiitis

**DOI:** 10.1371/journal.pone.0243572

**Published:** 2020-12-10

**Authors:** Hiroya Tanaka, Makoto Yamaguchi, Takayuki Katsuno, Hirokazu Sugiyama, Shiho Iwagaitsu, Hironobu Nobata, Hiroshi Kinashi, Shogo Banno, Takuji Ishimoto, Yasuhiko Ito

**Affiliations:** 1 Department of Nephrology and Rheumatology, Aichi Medical University, Nagakute, Japan; 2 Department of Nephrology, Suzuka General Hospital, Suzuka, Japan; 3 Department of Nephrology and Renal Replacement Therapy, Nagoya University Graduate School of Medicine, Nagoya, Japan; Nippon Medical School, JAPAN

## Abstract

Previous studies have evaluated the risk factors for relapse of antineutrophil cytoplasmic antibody (ANCA)-associated vasculitis (AAV) and the biomarkers of AAV for predicting relapse. However, little is known about the association between the presence of sinusitis and relapse and changes in the ANCA levels in AAV. This single-center, retrospective cohort study included 104 consecutive patients who were newly diagnosed with myeloperoxidase (MPO)-ANCA-positive microscopic polyangiitis (MPA) between 2006 and 2018 and were treated at the Aichi Medical University Hospital in Japan. The relationships between sinusitis and relapse of vasculitis and elevated MPO-ANCA levels were assessed using multivariate Cox proportional hazards models that were adjusted for clinically relevant factors. During the entire follow-up period (median, 24 months; interquartile range, 7–54 months), 93 (89.4%) patients achieved remission. After achieving remission, 38 (40.9%) patients experienced at least one relapse (13 [65.0%] in the sinusitis group; 25 [34.3%] in the non-sinusitis group). Sinusitis was identified as a significant predictor of relapse (adjusted hazard ratio: 2.41, 95% confidence interval [CI]: 1.19–4.88; P = 0.015). Furthermore, sinusitis was more likely to be associated with elevated MPO-ANCA levels (adjusted hazard ratio: 2.59, 95% CI: 1.14–5.92; P = 0.024). In conclusion, sinusitis was associated with a higher risk of relapse and elevated MPO-ANCA levels in MPA patients, suggesting that careful management may be required to reduce the risk of relapse in patients with sinusitis. Further studies are needed to elucidate the optimal treatment strategy for these patients.

## Introduction

Antineutrophil cytoplasmic antibody (ANCA)-associated vasculitis (AAV) is a disease characterized by small vessel inflammation and the presence of circulating ANCA. It includes granulomatosis with polyangiitis (GPA), microscopic polyangiitis (MPA), and eosinophilic granulomatosis with polyangiitis (EGPA) [1]. The accumulated clinical evidence on immunosuppressive treatment as an induction therapy for AAV has shown that remission and reduction of fatal organ damage are possible [2–5]. However, frequent relapses may occur in up to 50% of the patients [2–5]. It is important to identify modifiable risk factors for relapse and distinguish a biomarker that reliably predicts relapse, and this remains a research goal.

Clinical risk factors for AAV relapse have been identified in observational cohort studies [1], primarily on patients with GPA [1, 6], and include the proteinase 3-ANCA level [1, 6], upper respiratory tract disease [7], lung involvement [7], reduced serum creatinine levels at the time of presentation [6], chronic nasal *Staphylococcus aureus* carriage [1, 8, 9], and prior relapse [6]. However, it is unknown whether sinusitis is a risk factor for relapse in patients with MPA, who usually experience sinusitis less frequently as compared to patients with GPA [1–5].

ANCA has been proposed as a biomarker of impending AAV relapse [10]. Many physicians measure ANCA levels in clinical practice, and patients with increasing levels may require a more careful follow-up. Nevertheless, no previous studies have assessed the potential predictors for elevated ANCA levels.

We aimed to evaluate the relationship between sinusitis, the relapse thereof, and the increased myeloperoxidase (MPO)-ANCA levels in MPA patients in Japan.

## Materials and methods

### Ethical statements

The study protocol was approved by the Ethics Committee of the Aichi Medical University (approval number: 2018-H350; date: November 3, 2019). Due to the retrospective nature of the study, the need for patients’ informed consent was waived.

### Participants

**Fig 1:**
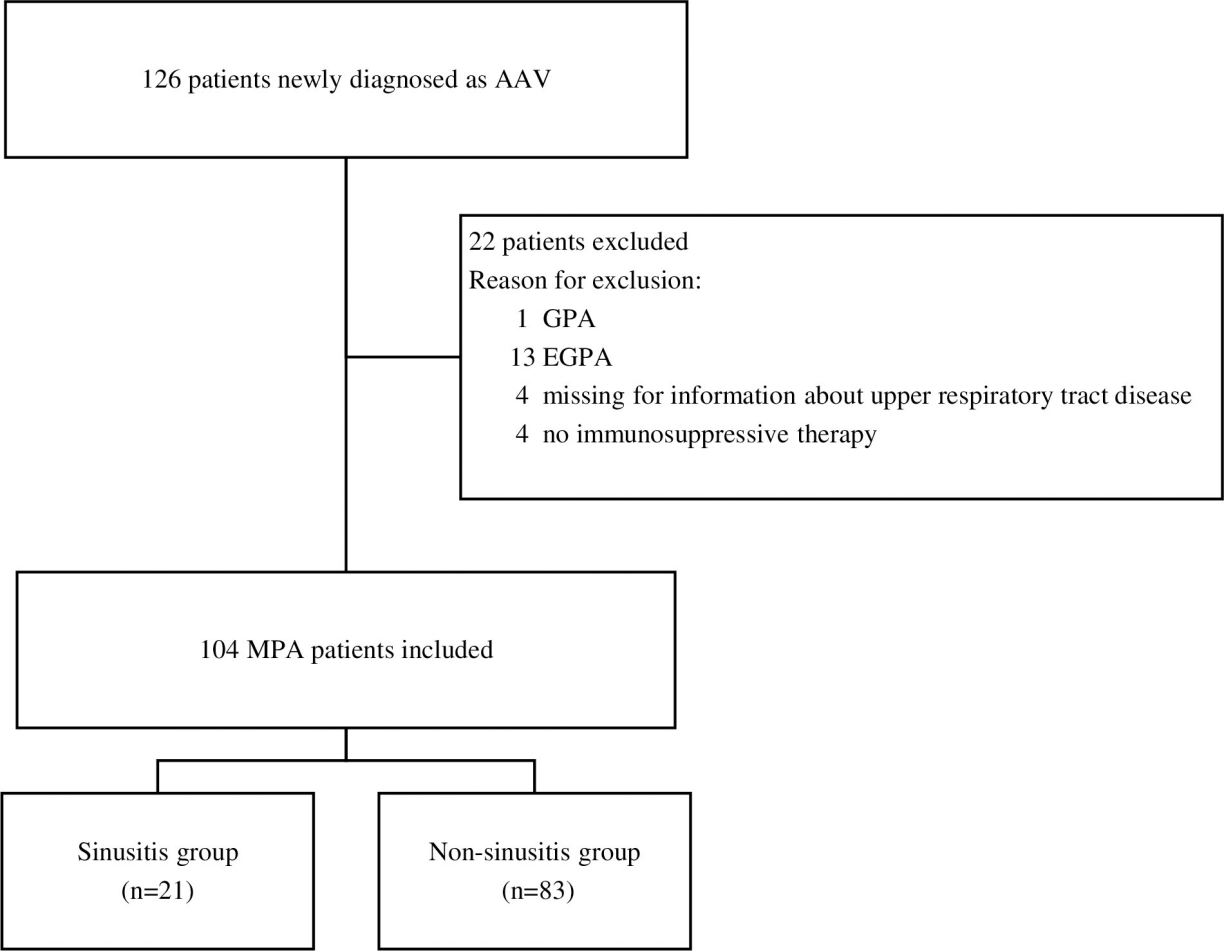


Between January 2006 and December 2018, 126 patients aged ≥20 years were newly diagnosed with AAV, including GPA, MPA, and EGPA, based on the European Medicines Agency algorithm [11], at the Nephrology and Rheumatology Center, Aichi Medical University, Japan. Twenty-two (17.5%) patients were excluded: 1 (0.8%) and 13 (10.3%) patients had GPA and EGPA, respectively; immunosuppressive therapy was not used in four (3.2%) patients, and no data on sinusitis were available at presentation in four (3.2%) patients. Finally, 104 (82.5%) consecutive patients with MPA who received immunosuppressive therapy and were followed up until April 2020 were included. These patients were categorized into the following two groups: the sinusitis group (comprising of 21 [20.1%] patients with sinusitis at baseline) and the non-sinusitis group (comprising of 83 [79.8%] patients without sinusitis at baseline) (Fig 1).

All data were fully anonymized (S1 Table).

### Covariates

The clinical characteristics at the time of initiating immunosuppressive therapy (baseline data) were collected from the patients’ medical records, as previously described [12, 13]. We obtained the following patient data: the age; sex; serum creatinine levels; estimated glomerular filtration rate (eGFR; mL/min/1.73 m^2^ = 194×Scr^_1.094^×age^_0.287^×0.739 [if female]) [14]; C-reactive protein levels; Birmingham Vasculitis Activity Score (BVAS) 2003 [15]; organ involvement, anti-MPO-ANCA levels; immunosuppressive treatment; induction immunosuppressive therapy including the use of methylprednisolone pulse therapy (0.5 or 1.0 g/d for 3 consecutive days), intravenous cyclophosphamide (CYC), and intravenous rituximab (RTX); initial dose of prednisolone (PSL) (mg/kg/day); and maintenance therapy including CYC, azathioprine (AZA), methotrexate, mizoribine, and RTX.

Data on the immunosuppressive treatment regimen at the time of and after relapse were obtained, including the dose of PSL (mg/day) and the use of immunosuppressants.

The primary exposure of interest was sinusitis at presentation, which was defined by the presence of specific findings on computed tomography (CT), as previously described [16].

### Evaluation of the ANCA levels

Direct antigen-specific enzyme-linked immunosorbent assays for MPO-ANCA with serially diluted serum were performed, as previously described [17]; the samples were diluted 1:101 (Medical and Biological Laboratories Co., Ltd., Nagoya, Japan) or 1:500 (Nipro Medical Corporation, Osaka, Japan).

A physician evaluated the ANCA levels during each patient’s visit, which usually occurred monthly. Negative conversion of ANCA was defined as the disappearance of ANCA. An increase in the ANCA level was defined as a positive conversion of ANCA in patients who achieved negative conversion.

### Outcomes

The outcome measures of interest in the present study consisted of the time from remission to first relapse of MPA and the time from negative to positive conversion of MPO-ANCA levels. Remission was defined as the absence of clinical signs and symptoms of active vasculitis (BVAS = 0) for >2 months. Relapse was defined as the recurrence or new onset of clinical signs and symptoms of active vasculitis, as previously described [18].

Other outcome data included death, end-stage renal disease (ESRD) requiring permanent dialysis, and hospitalization because of infection.

Patients were followed up until April 2020, either until the last day of examination at our hospital or until death before April 2020.

### Statistical analyses

Comparisons of clinical characteristics between the two groups were performed using the Wilcoxon rank-sum test or the Fisher’s exact test, as appropriate. The cumulative probabilities of the first relapse were estimated using the Kaplan–Meier method and compared using the log-rank test. Potential covariates were examined using univariate and multivariate Cox proportional hazard models to identify the prognostic factors independently associated with relapse; the complete data of 93 (89.4%) patients who achieved remission were available for these univariate and multivariate analyses. Furthermore, we also used univariate and multivariate Cox proportional hazard models for evaluating the predictors of elevated MPO-ANCA levels after including 74 (71.2%) patients who achieved negative conversion of MPO-ANCA levels. Data were adjusted for baseline characteristics, including the age, sex, lung involvement, serum creatinine level, and sinusitis, based on previous reports [1, 6–9]. The proportionality assumption was tested using scaled Schoenfeld residuals. P-values <0.05 were considered significant. All statistical analyses were performed using JMP version 14.0.0 (SAS Institute, Cary, NC, USA) and STATA version 13.0 (Stata Corp LP, College Station, TX, USA).

## Results

The clinical characteristics of the 21 (20.2%) and 83 (79.8%) patients with and without sinusitis are listed in Table 1. The sinusitis group had a higher proportion of ear and throat involvement than the non-sinusitis group (P = 0.002). The other factors at baseline did not differ significantly between the two groups. Furthermore, no significant difference in the induction immunosuppressive treatment was found between the two groups; approximately 15% of the patients in both groups received intravenous CYC or RTX as induction therapy. Regarding maintenance treatment, approximately two-thirds of the patients in both groups received glucocorticoid monotherapy, and there were no significant differences in the treatment with other immunosuppressive agents between the groups (P = 0.394).

**Table 1:** Clinical characteristics of 104 patients with MPO-ANCA-positive MPA.

	Non-sinusitis group (n = 83)	Sinusitis group (n = 21)	*P value*
**Baseline characteristics**			
Age (years)	74 (68–78)	69 (64–77)	0.095
Males	45 (54.2)	12 (57.1)	0.810
Serum creatinine level (mg/dL)	1.4 (0.9–3.9)	1.1 (0.7–2.1)	0.198
eGFR [mL/min/1.73 m^2^]	46 (27–82)	47 (26–93)	0.853
CRP level (mg/dL)	3.6 (1.2–9.5)	6.5 (1.1–10.1)	0.848
BVAS	14 (11–15)	12 (11–18)	0.807
**Organ involvement**			
General	81 (97.6)	21 (100)	0.473
Cutaneous	5 (6.0)	0 (0.0)	0.249
Ear and throat	3 (3.6)	5 (23.8)	0.002
Chest	27 (32.5)	6 (28.6)	0.728
Cardiovascular	0 (0)	0 (0)	1.000
Abdominal	2 (2.4)	0 (0)	0.473
Renal	67 (80.7)	16 (76.2)	0.644
Nervous system	12 (14.5)	5 (23.8)	0.301
**Induction immunosuppressive therapy**			
Intravenous cyclophosphamide	9 (10.8)	1 (4.8)	0.398
Rituximab	6 (7.2)	2 (9.5)	0.724
Use of mPSL pulse therapy	41 (49.4)	7 (33.3)	0.187
Initial dose of prednisolone (mg/day)	30 (30–40)	30 (30–40)	0.257
**Maintenance immunosuppressive therapy**			0.394
Glucocorticoid monotherapy	59 (71.1)	14 (66.7)	
Oral cyclophosphamide	2 (2.4)	0 (0)	
Azathioprine	16 (19.3)	5 (23.8)	
Methotrexate	0 (0)	1 (4.8)	
Mizoribine	2 (2.4)	0 (0)	
Rituximab	4 (4.8)	1 (4.8)	
**Outcome**			
Remission	73 (88.0)	20 (95.2)	0.332
Relapse	25 (34.3)	13 (65.0)	0.013
BVAS at relapse	8 (6–9)	8 (7–9)	0.621
Organ involvement at relapse			
General	22 (88.0)	10 (76.9)	0.374
Cutaneous	1 (4.0)	0 (0)	0.465
Ear and throat	1 (4.0)	2 (15.4)	0.217
Chest	1 (4.0)	2 (15.4)	0.217
Cardiovascular	0 (0)	0 (0)	1.000
Abdominal	0 (0)	0 (0)	1.000
Renal	8 (32.0)	5 (38.5)	0.690
Nervous system	2 (8.0)	4 (30.7)	0.068
HD	18 (21.7)	4 (19.1)	0.791
Death	19 (22.9)	3 (14.3)	0.388
Hospitalization due to infection	26 (31.3)	4 (19.1)	0.267
**Changes in ANCA levels**			
Negative conversion in ANCA levels	57 (68.7)	17 (81.0)	0.267
Increase of ANCA levels	16 (28.1)	14 (82.4)	<0.001
Observation period (months)	16 (7–34)	17 (5–30)	0.749

Continuous data are presented as a median (interquartile range), and categorical data are expressed as a number (proportion).

Abbreviations: ANCA, anti-neutrophil cytoplasmic antibody; MPA, microscopic polyangiitis; BVAS, Birmingham Vasculitis Activity Score; CRP, C-reactive protein; eGFR, estimated glomerular filtration rate; mPSL, methylprednisolone; HD, hemodialysis.

Except for one patient in the non-sinusitis group who had an allergic reaction to trimethoprime-sulfamethoxazole, the 103 (99.0%) patients included had been prescribed trimethoprime-sulfamethoxazole routinely for the prevention of Pneumocystis pneumonia.

Furthermore, no patient in the sinusitis group presented with typical clinical symptoms associated with bacterial sinusitis, such as purulent nasal discharge, nasal stuffiness, and facial pain; therefore, antibiotics were not prescribed for sinusitis during the observation period.

### Remission and relapse

During a median observational period of 24 months (interquartile range [IQR]: 7–54 months), 93 (89.4%) patients, from the entire cohort (sinusitis group: 20 [95.2%] patients; non-sinusitis group: 73 [88.0%] patients) achieved remission (P = 0.332) (Table 1). After remission, 38 (40.9%) patients (sinusitis group: 13 [65.0%] patients; non-sinusitis group: 25 [34.3%] patients) relapsed at least once during the observational period (P = 0.013) (Table 1). No significant differences in the BVAS and organ involvement at relapse were found between the two groups (Table 1). At relapse, as well as at the time of MPA diagnosis, only three (12.0%) patients in the non-sinusitis group and two (15.4%) patients in the sinusitis group had been evaluated by sinus CT. Although no findings indicative of sinusitis were observed in the non-sinusitis group, two (100%) patients in the sinusitis group presented with CT findings of sinusitis asymptomatically, which had not changed significantly from the time of MPA diagnosis.

**Fig 2:**
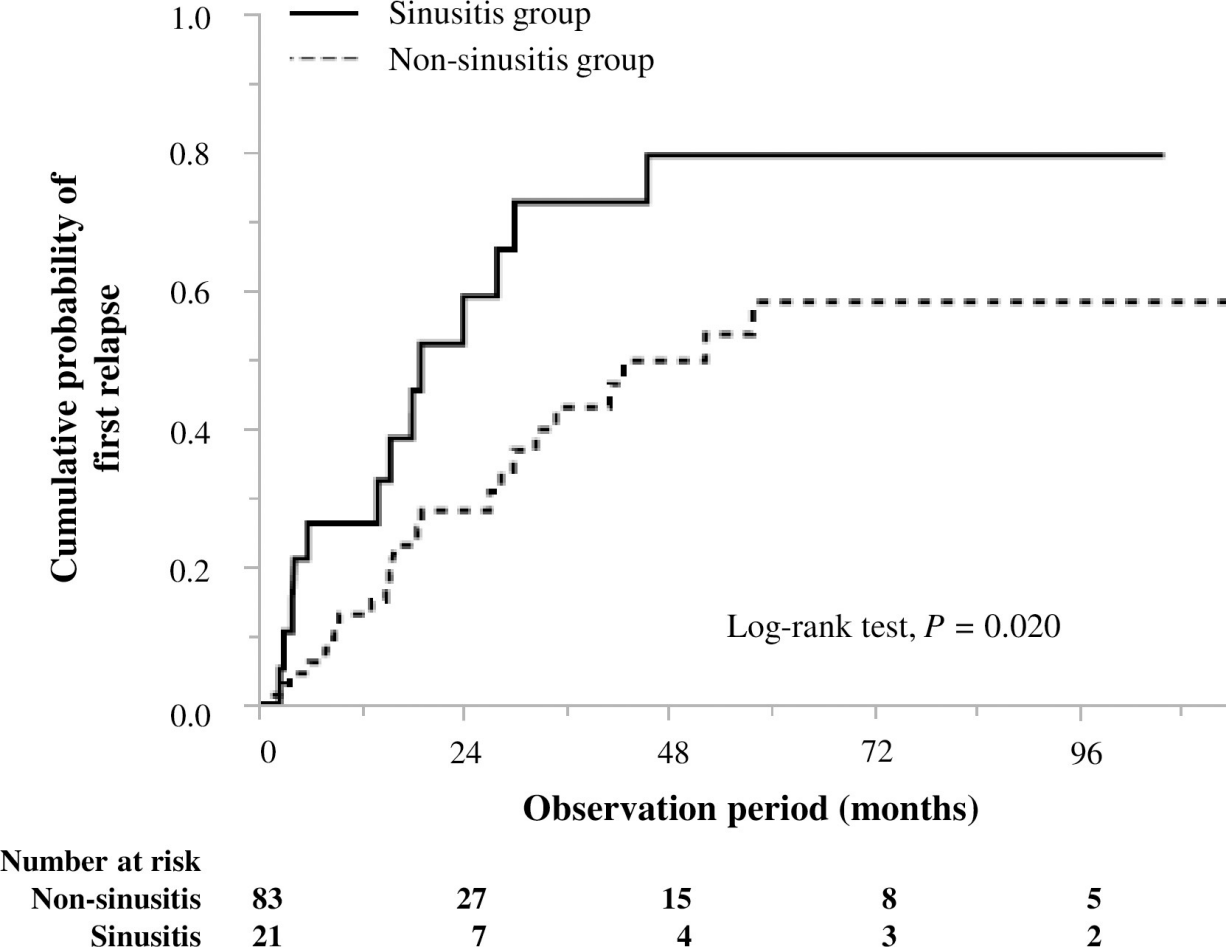


Cumulative probabilities of the first relapse at 1, 2, and 5 years were 0.26, 0.59, and 0.80, respectively, in the sinusitis group and 0.13, 0.28, and 0.58, respectively, in the non-sinusitis group, suggesting that the incidence of the first relapse was significantly higher in the sinusitis group than in the non-sinusitis group (log-rank test: P = 0.020) (Fig 2).

### Predictors of relapse

Table 2 shows the association between sinusitis and relapse in univariate and multivariate Cox proportional hazard models. In the univariate model, the presence of sinusitis was associated with a higher risk of relapse. Multivariate models also identified sinusitis as a significant predictor of relapse (adjusted hazard ratio [HR]: 2.41, 95% confidence interval [CI]: 1.19–4.88, P = 0.015).

**Table 2:** Predictors of the first relapse.

	Univariate model		Multivariate model	
	HR (95% CI)	*P* value	HR (95% CI)	*P* value
**Age (per 10 years)**	0.97 (0.76–1.29)	0.826	1.03 (0.79–1.39)	0.850
**Male (vs. female)**	1.07 (0.56–2.04)	0.849	1.13 (0.58–2.20)	0.722
**Lung involvement**	1.17 (0.58–2.33)	0.660	1.23 (0.61–2.48)	0.570
**Serum creatinine (per 1.0 mg/dL)**	0.92 (0.79–1.05)	0.256	0.91 (0.78–1.03)	0.161
**Sinusitis**	2.18 (1.11–4.30)	0.024	2.41 (1.19–4.88)	0.015

Abbreviations: HR, hazard ratio; CI, confidence interval; vs., versus.

Data are presented as HRs, 95% CIs, and *P* values from the Cox proportional hazard regression analyses.

Data are adjusted for baseline characteristics including age, sex, lung involvement, serum creatinine level, and sinusitis.

### Immunosuppressive treatment at relapse

The median dose of PSL (mg/day) at the time of relapse did not differ significantly between the two groups (sinusitis group: 9 mg/day [IQR: 7–12 mg/day]; non-sinusitis group: 8 mg/day [IQR: 4–10 mg/day]; P = 0.290). The proportion of use of an immunosuppressive agent combined with glucocorticoid therapy at the time of relapse was not significantly different between the two groups as well (sinusitis group: 4 [30.8%] patients; non-sinusitis group: 12 [48.0%] patients; P = 0.260).

At the time of relapse, the steroid dose was increased in all patients, and the immunosuppressive agent was changed if required: The PSL dose was increased from 9 (IQR: 7–12) to 20 (IQR: 15–30) mg/day in the sinusitis group and from 8 (IQR: 2–10) to 20 (IQR: 14–20) mg/day in the non-sinusitis group. Additionally, the immunosuppressive agent was changed in six patients (mizoribine was changed to AZA in one patient, AZA was initiated in two patients, intravenous CYC was initiated in two patients, and intravenous RTX was initiated in one patient).

### Change in the MPO-ANCA levels

Overall, 74 (71.2%) patients achieved negative conversion of their MPO-ANCA levels (17 [81.0%], sinusitis; 57 [68.7%], non-sinusitis; P = 0.267) (Table 1), and in the remaining 31 (29.8%) patients, the MPO-ANCA levels decreased compared to the baseline levels, regardless of whether the negative conversion was achieved or not. Of the 74 patients with negative conversion of MPO-ANCA levels, 30 (40.5%) patients had increased MPO-ANCA levels (14 [82.4%], sinusitis; 16 [28.1%], non-sinusitis; P < 0.001) (Table 1). Among patients who achieved remission, 27 (90.0%) patients had relapse after confirming increased MPO-ANCA levels. The median time from an increase in MPO-ANCA levels to relapse was 1.8 months (IQR, 0.6–5.0 months).

### Predictors of increased MPO-ANCA levels

Table 3 shows the association of clinical characteristics with elevated MPO-ANCA levels in the univariate and multivariate Cox proportional hazard models. In the univariate model, the presence of sinusitis was associated with a higher risk of elevated ANCA levels. Multivariate models also identified sinusitis as a significant predictor of elevated ANCA levels (adjusted HR: 2.59, 95% CI: 1.14–5.92; P = 0.024).

**Table 3:** Predictors of elevated MPO-ANCA levels.

	Univariate model		Multivariate model	
	HR (95% CI)	*P* value	HR (95% CI)	*P* value
**Age (per 10 years)**	0.79 (0.61–1.05)	0.103	0.93 (0.68–1.32)	0.649
**Male (vs. female)**	1.21 (0.59–2.50)	0.606	0.91 (0.43–1.93)	0.810
**Lung involvement**	0.72 (0.32–1.62)	0.431	1.04 (0.44–2.46)	0.935
**Serum creatinine (per 1.0 mg/dL)**	0.95 (0.79–1.10)	0.527	0.95 (0.80–1.09)	0.516
**Sinusitis**	2.83 (1.38–5.82)	0.005	2.59 (1.14–5.92)	0.024

Abbreviations: HR, hazard ratio; CI, confidence interval; vs., versus; MPO, myeloperoxidase; ANCA, anti-neutrophil cytoplasmic antibody.

Data are presented as HRs, 95% CIs, and *P* values from the Cox proportional hazard regression analyses.

Data are adjusted for baseline characteristics including age, sex, lung involvement, serum creatinine level, and sinusitis.

### Adverse events

During the median observational period of 24 months (IQR: 7–54 months), 22 (21.2%) patients required dialysis (sinusitis group: 4 [19.1%] patients, non-sinusitis group: 18 [21.7%] patients; P = 0.791) and 22 (21.2%) patients died (sinusitis group: 3 [14.3%] patients, non-sinusitis group: 19 [22.9%] patients; P = 0.388). No significant differences in the rates of hospitalization for infection were observed between the two groups (sinusitis group: 4 [19.1%] patients, non-sinusitis group: 26 [31.3%] patients; P = 0.267) (Table 1).

## Discussion

The study identified a significant association between the presence of sinusitis and a relapse in patients with MPA and elevated MPO-ANCA levels, even after adjusting for clinically relevant risk factors. This suggests that sinusitis may be a predisposing factor for relapse in patients with MPA. Ours is the first study to focus on the clinical effect of sinusitis on AAV relapse and changes in the ANCA levels. Our results may prove useful for detecting patients who require careful monitoring for relapse and may help develop the optimal strategy for preventing relapse.

Only one study that included two national cohorts [7] consisting predominantly of GPA patients has assessed the relationship between upper respiratory tract disease (defined as sinusitis or otitis media) and relapse in AAV. However, the results were inconsistent in each cohort: In one community-based American cohort of 347 patients with newly diagnosed AAV, upper respiratory tract disease was identified as a significant risk factor for relapse in AAV (HR: 1.58, 95% CI: 1.00–2.48; P = 0.048). In contrast, in another community-based French cohort of 434 patients, upper respiratory tract disease was not significantly associated with relapse (HR: 0.96, 95% CI: 0.67–1.38; P = 0.820). Several points should be considered when interpreting the differences in these results. First, these two cohorts differed in the prevalence of upper respiratory tract disease (US cohort: 31%, French cohort: 62%). Therefore, this difference in the clinical characteristics between the two cohorts may influence the results on the clinical impact of upper respiratory tract disease on the risk of relapse in AAV. Second, it remains to be confirmed whether the result is applicable to patients with MPA, because these studies mainly included participants with GPA.

Meanwhile, although several cohort studies predominantly focusing on patients with MPA, have been performed [19, 20], no study has assessed sinusitis as a covariate in multivariate models for evaluating relapse risk. Therefore, the clinical impact of sinusitis on AAV is unknown.

With respect to a biomarker of AAV for predicting relapse, a meta-analysis included mainly patients with GPA and showed that persistent ANCA positivity or the development of positivity following a negative test showed modest predictive power, with a positive likelihood of 1.97 (95% CI: 1.43–2.70) [21]. In addition, a prospective study on a Japanese cohort consisting mainly of patients with MPA revealed that reappearance of MPO-ANCA had a significant association with a subsequent relapse, with an OR of 26 (95% CI: 8.2–101.0) [22].

Considering these results, standard ANCA measurements during remission remain a feature of clinical practice for many physicians, and patients with increasing ANCA levels may warrant more careful follow-up, although it remains controversial whether an immediate change of therapy is necessary [23, 24]. However, no previous report has focused on the predictor for a rise in ANCA levels. In the present study, we identified a significant relationship between sinusitis and the subsequent increase in MPO-ANCA levels, suggesting that sinusitis itself may be a trigger for ANCA production and a predisposing factor for relapse in AAV.

The precise mechanism of the clinical effect of sinusitis on relapse in AAV is unclear; one of the biologically plausible hypotheses is infection. Several cohort studies have demonstrated a higher rate of *S*. *aureus* nasal carriage among GPA patients with relapse, thereby implicating *S*. *aureus* infection as a possible inciting factor for relapse of GPA [1, 8, 9]. Infection may play a role in relapse through molecular mimicry, wherein antibodies to microbial antigens cross-react with neutrophil antigens, or through the development of antibodies to complementary peptides and the subsequent host immune response to these anti-complementary PR3 antibodies [25, 26]. Additionally, in our study, sinusitis might have triggered a relapse of AAV through infection, even without the presence of obvious clinical symptoms associated with bacterial sinusitis. Further studies are essential to clarify the association between sinusitis and relapse by assessing these possible mechanisms.

The present study has some limitations. First, because of the retrospective nature of the study, the intervals between consecutive ANCA level measurements were not equal across the cohort, and the immunosuppressive treatment regimen was not standardized. Second, we did not focus on the clinical effect of changes in the ANCA levels on AAV relapse in patients who did not achieve negative conversion of ANCA; this requires further studies. Third, the study did not assess the CT findings of sinusitis after immunosuppressive treatment was initiated. Thus, the state of sinusitis was not evaluated precisely by CT at remission and/or relapse, and it is unknown whether sinusitis was due to vasculitis, infection, or coexistence. Fourth, the study included only patients with MPA, and the sample size was small; therefore, it is uncertain whether our results are applicable to all types of AAV. Our findings require validation in a large well-designed cohort study. Furthermore, the short observational period of the present study (24 months [IQR: 7–54 months) hindered a meaningful analysis of an association between sinusitis and relapse in AAV, and this association should be ascertained in a cohort with a longer observational period. Fifth, the European Medicines Agency algorithm [11] defined "chronic sinusitis" as a surrogate marker for GPA, which is defined as a state of sinusitis lasting for more than 3 months. However, in the present study, all sinusitis patients only presented with findings of sinusitis on CT scans, and all of these patients were asymptomatic; therefore, we did not classify them as having GPA.

Furthermore, from the results of the one-time CT scan, it was difficult to ascertain whether these cases had acute sinusitis or chronic sinusitis. If some of the patients had chronic sinusitis, they could be reconsidered as having GPA (MPO-positive). This point should be interpreted cautiously, and it should be validated in other studies. Lastly, mainly steroid monotherapy was used, as most patients were treated with corticosteroids alone; however, a few patients were treated with a combination of glucocorticoids and either CYC or RTX, according to either the American College of Rheumatology or the European League Against Rheumatism recommendations for the management of AAV [4]. Hence, our results should be interpreted cautiously.

## Conclusion

This study identified that sinusitis carries a higher risk of relapse in AAV and that ANCA levels increase in patients with MPO-positive MPA, suggesting that careful management may be required in patients with sinusitis at presentation to reduce the risk of AAV relapse.

## Supporting information

S1 Table The anonymized data set of the present study. (XLSX)
